# Bryozoans are returning home: recolonization of freshwater ecosystems inferred from phylogenetic relationships

**DOI:** 10.1002/ece3.1352

**Published:** 2014-12-21

**Authors:** Nikola Koletić, Maja Novosel, Nives Rajević, Damjan Franjević

**Affiliations:** 1 Institute for Research and Development of Sustainable EcosystemsJagodno 100a, 10410, Velika Gorica, Croatia; 2 Department of Biology, Faculty of Science, University of ZagrebRooseveltov trg 6, 10000, Zagreb, Croatia

**Keywords:** COI, Gymnolaemata, ITS2, Phylactolaemata, rRNA genes

## Abstract

Bryozoans are aquatic invertebrates that inhabit all types of aquatic ecosystems. They are small animals that form large colonies by asexual budding. Colonies can reach the size of several tens of centimeters, while individual units within a colony are the size of a few millimeters. Each individual within a colony works as a separate zooid and is genetically identical to each other individual within the same colony. Most freshwater species of bryozoans belong to the Phylactolaemata class, while several species that tolerate brackish water belong to the Gymnolaemata class. Tissue samples for this study were collected in the rivers of Adriatic and Danube basin and in the wetland areas in the continental part of Croatia (Europe). Freshwater and brackish taxons of bryozoans were genetically analyzed for the purpose of creating phylogenetic relationships between freshwater and brackish taxons of the Phylactolaemata and Gymnolaemata classes and determining the role of brackish species in colonizing freshwater and marine ecosystems. Phylogenetic relationships inferred on the genes for 18S rRNA, 28S rRNA, COI, and ITS2 region confirmed Phylactolaemata bryozoans as radix bryozoan group. Phylogenetic analysis proved Phylactolaemata bryozoan's close relations with taxons from Phoronida phylum as well as the separation of the Lophopodidae family from other families within the Plumatellida genus. Comparative analysis of existing knowledge about the phylogeny of bryozoans and the expansion of known evolutionary hypotheses is proposed with the model of settlement of marine and freshwater ecosystems by the bryozoans group during their evolutionary past. In this case study, brackish bryozoan taxons represent a link for this ecological phylogenetic hypothesis. Comparison of brackish bryozoan species *Lophopus crystallinus* and *Conopeum seurati* confirmed a dual colonization of freshwater ecosystems throughout evolution of this group of animals.

## Introduction

Bryozoans are one of the most enigmatic groups in the animal kingdom regarding their insufficiently known evolutionary relationships. In the 20th century, Bryozoa phylum was divided into three classes – Phylactolaemata, Stenolaemata, and Gymnolaemata (Woollacott and Zimmer 1977). Phylactolaemata contains approximately 80 freshwater species (Wood 2002; Massard and Geimer 2008), Stenolaemata comprises approximately 700 marine species, while Gymnolaemata includes approximately 5000 species (Gordon 2003) most of which are marine species, while some live in brackish waters.

The study of the nucleotide composition of 28S rRNA gene supported the close relationship of Phylactolaemata and Stenolaemata, while the analysis of the 18S rRNA and COI gene classified Phylactolaemata as the radix group within the Bryozoa phylum (Fuchs et al. 2009). Monophyletic origin of bryozoans has been proved by molecular approach with the representatives of Phoronida and Brachiopoda (Fuchs et al. 2009). Phylogenetic studies show a closer affinity of Phylactolaemata to Phoronida than with other classes of Bryozoa (Mundy et al. 1981) and closer evolutionary relatedness between Stenolaemata and Gymnolaemata classes (Waeschenbach et al. 2012). Molecular phylogenies of bryozoans have been published using a single gene for small subunit of nuclear ribosomal RNA gene (18S rRNA) (Jiao et al. 2009; Tsyganov-Bodounov et al. 2009), large mitochondrial ribosomal subunit (16S rRNA) (Dick et al. 2000; Hao et al. 2005), large subunit of nuclear ribosomal RNA gene (28S rRNA), and the bar coding region of mitochondrial cytochrome c oxidase subunit 1 (COI) (Fuchs et al. 2009), or by combining nuclear genes and the bar coding region of COI (Knight et al. 2011).

Until now, over 16 000 species of fossil bryozoans have been discovered. The oldest discovered bryozoans with the mineral shell form are about 500 million years old, but it is assumed that the bryozoans with softshells emerged earlier. Fossil representatives of Phylactolaemata bryozoans are very rare. Findings of freshwater taxons are scarce, while rare specimens of bryozoans with softshells are found in 260-million-year old layers, from the Late Permian (Dewel et al. 2002). The lack of fossil remains of taxons from the Phylactolaemata class is explained by the fact that all members of this class, unlike Gymnolaemata and Stenolaemata, are entirely freshwater organisms and are built exclusively of soft tissue without any mineral compound. Freshwater and brackish bryozoan colonies are products of asexual reproduction using statoblasts (Wood and Okamura 2005). Statoblasts are the main vectors of dispersal, and they resist desiccation and allow overwintering. Fossil records of Phylactolaemata bryozoans are limited to sporadic preservation of statoblasts. The earliest fossil evidence comes from the Upper Permian ∼260 Ma Plumatellidae (Vinogradov 1996) and from the Upper Triassic ∼215 Ma Pectinatellidae (Kohring and Hörnig 2002).

The focus of this research was on freshwater and brackish bryozoan taxons from the Phylactolaemata and Gymnolaemata classes and overlapping their phylogeny with known genetic relationships of marine and brackish taxons from the Gymnolaemata class. Representatives of Phoronida and Brachiopoda were chosen as out-groups. The comparison of evolutionary relatedness between freshwater and brackish taxons with out-group taxons was made on four different phylogenetic markers (18S, 28S, ITS2, and COI) using two different methods of phylogenetic inference (maximum likelihood and Bayesian analysis). The goal of the research was to gain more congruent phylogenetic relationships and evolutionary history of freshwater and brackish bryozoans taxons given the existing literature data and experimental data obtained for this study.

## Methods

### Sampling

**Figure 1:**
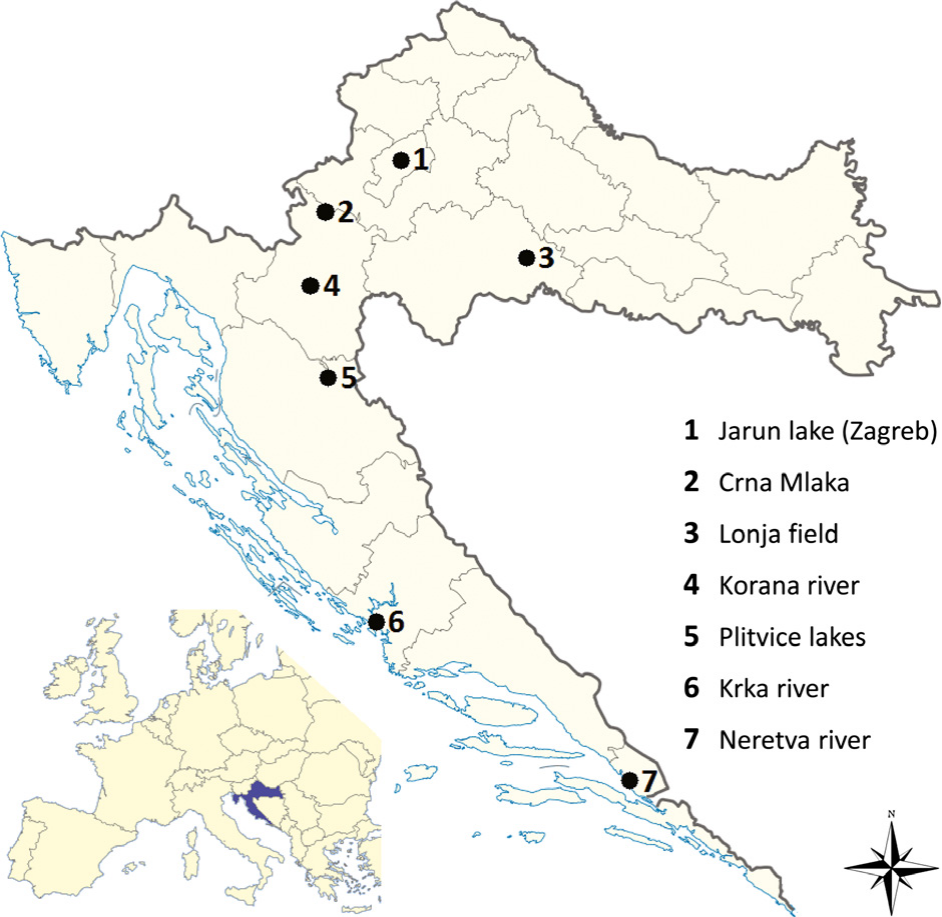
Sampling locations in Croatia where bryozoan taxons were collected. At locations in the Krka River and the Neretva River, the tissue samples of brackish taxons were collected.

Tissue samples of bryozoan freshwater and brackish colonies were collected from 2008 till 2013 in the rivers of Adriatic and Danube basin and in the wetland areas in the continental part of Croatia (Europe), at seven locations, namely Jarun Lake in Zagreb, wetlands Crna Mlaka and Lonja Field, the Korana River, the Plitvice Lakes, the Krka River, and the Neretva River (Fig.1). Altogether, twelve taxons of bryozoans were morphologically identified (Garašić 2009; Janjiš 2009, Wöss and Novosel 2013). Two of collected samples belong to the Gymnolaemata class (*Conopeum seurati* and *Paludicella articulata*), while ten of them belong to the Phylactolaemata class (*Cristatella mucedo, Fredericella sultana, Lophopus crystallinus, Hyalinella punctata, Plumatella casmiana, Plumatella emarginata, Plumatella fruticosa, Plumatella fungosa, Plumatella geimermassardi,* and *Plumatella repens*). From river brackish water, two taxons were identified (*Conopeum seurati* and *Lophopus crystallinus*), while other ten were collected in exclusively freshwater environment (Koletić 2014). The material was fixed and stored in 95% ethanol until DNA extraction (Wood 2005). Tissue samples of each specific taxon were used for further phylogenetic analysis.

### DNA extraction, PCR, and sequencing

Total genomic DNA was extracted from ethanol-preserved specimens using the DNeasy Blood & Tissue kit (QIAGEN), following the manufacturer protocol. Four different phylogenetic markers have been selected for this study: nuclear genes for 18S rRNA, 28S rRNA, and ITS2 region and mitochondrial gene for cytochrome c oxidase subunit 1 (COI). PCR analysis was carried out in 25 *μ*L reaction volumes containing TopTaq Master Mix kit (QIAGEN), 1*μ*L of DNA, 1 *μ*L of 10 *μ*mol/L of each primer, and 22 *μ*L of distilled RNA-free water. Used primers are listed in Table1 and PCR cycling conditions in Table2. All PCR products were tested for the presence of amplified products on 1% agarose gels. PCR products were purified using MinElute Reaction Cleanup kit (QIAGEN). Sequences obtained after using commercial sequencing service of Macrogen Company (Netherlands) were deposited in GenBank.

**Table 1:** Primers used for PCR amplification and sequencing

DNA region	Primer name	Primer sequence	Reference
18S	5F	5′-GCGAAAGCATTTGCCAAGAA-3′	Giribet et al. (1996)
	9R	5′-GATCCTTCCGCAGGTTCACCTAC-3′	
28S	1F	5′-ACCCGCTGAATTTAAGCAT-3′	Féral et al. (1994)
	3R	5′-CACCTTGGAGACCTGCT-3′	
ITS2	ITS2rück	5′-CGGGGATTCGGCGCTGGG-CTCTTCCC-3′	Ohst (2008)
	ITS2hin	5′-GGATCACTCGGCTCGTGCGTCGATGAAG-3′	
COI	LCO-1490	5′-GGTCAACAAATCATAAAGATATTGG-3′	Folmer et al. (1994)
	HCO-2198	5′-TAAACTTCAGGGTGACCAAAAAATCA-3′	

**Table 2:** The PCR reaction conditions for the individual genes. The cycle of denaturation, annealing, and elongation was repeated 35 times

Reaction conditions	18S and ITS2		28S		COI	
	Temp.	Duration	Temp.	Duration	Temp.	Duration
Initial denaturation	94°C	3 min	94°C	3 min	94°C	3 min
Denaturation	94°C	30 sec	94°C	30 sec	94°C	30 sec
Annealing	60°C	30 sec	50°C	45 sec	55°C	30 sec
Elongation	72°C	1 min	72°C	1 min	72°C	1 min
Final elongation	72°C	10 min	72°C	10 min	72°C	10 min
Final hold	4°C	∞	4°C	∞	4°C	∞

### Phylogenetic analysis

The obtained nucleotide sequences of freshwater and brackish taxons from this case study were analyzed together with the available GenBank sequences of marine bryozoans and out-group taxons (Table3). The sequences were aligned using ClustalX 2.0 (Thompson et al.^1997^), and further editing was performed using BioEdit 7.0.9 (Hall 1999). Phylogenetic analyses were performed with maximum likelihood method (ML) and Bayesian analysis (BA). ML trees estimation was performed using PAUP* 4.0b10 (Swofford 2001). Bayesian analyses were performed using MrBayes3.1.2 (Huelsenbeck and Ronquist 2001). For the ML and BA analyses, MrMTgui 1.0 (Nuin 2005) and MrModeltest 3.7 (Posada and Crandall 1998) were used to search for best-fit models for the partitioned data sets; COI followed MIT + I + G model, 18S rDNA followed TrN + I + G, while 28S rDNA and ITS2 region followed TrN + G model under Akaike information criterion (AIC). Phylogenetic trees were estimated using bootstrap analysis (Felsenstein^1985^).

**Table 3:** Species used in this study with corresponding accession numbers

Phylum/class	Family	Species name	18S accession no.	28S accession no.	COI accession no.	ITS2 accession no.
Bryozoa						
Phylactolaemata	Cristatellidae	*Cristatella mucedo*	DQ221750	JN681027	FJ196106	EU377582
	Fredericellidae	*Fredericella sultana*	DQ221751	–	–	EU377581
	Lophopodidae	*Asajirella gelatinosa*	DQ221753	FJ196153	FJ196096	–
		*Lophopus crystallinus*	KJ024817^*^	JN681028	FJ196107	KJ024833^*^
	Pectinatellidae	*Pectinatella magnifica*	FJ409600	FJ196151	FJ196095	–
	Plumatellidae	*Gelatinella toanensis*	–	FJ196140	FJ196082	–
		*Hyalinella punctata*	KJ024819^*^	–	KJ024814^*^	KJ024836^*^
		*Plumatella bombayensis*	–	JN681032	–	–
		*Plumatella casmiana*	KJ024818^*^	KJ024827^*^	KJ024813^*^	KJ024834^*^
		*Plumatella emarginata*	KJ024816^*^	KJ024826^*^	KJ024812^*^	KJ024832^*^
		*Plumatella fungosa*	DQ221748	–	–	–
		*Plumatella geimermassardi*	JN680932	–	–	EU377578
		*Plumatella reticulata*	DQ530349	–	–	–
		*Plumatella repens*	KJ024815^*^	KJ024829^*^	KJ024811^*^	KJ024831^*^
		*Plumatella rugosa*	JN680931	–	–	–
		*Plumatella vaihiriae*	–	JN681031	–	EU377577
		*Stephanella hina*	JN680924	–	–	–
Gymnolaemata	Bugulidae	*Bicellariella ciliata*	–	FJ196157	–	–
		*Bugula plumosa*	JN680951	JN681045	–	–
	Candidae	*Caberea lata*	–	–	HQ896153	–
		*Scrupocellaria scruposa*	–	–	FJ196098	–
		*Tricellaria occidentalis*	–	–	HQ896152	–
	Electridae	*Conopeum seurati*	–	–	–	KJ024835^*^
		*Electra pilosa*	–	–	FJ196089	–
	Flustridae	*Flustra foliacea*	–	FJ196139	–	–
	Hippothoidae	*Celleporella hyalina*	–	FJ196137	–	–
	Membranipoidae	*Membranipora membranacea*	–	–	FJ196092	–
		*Membranipora serrilamella*	–	–	HQ896178	–
	Reteporellidae	*Reteporella beaniana*	–	–	FJ196084	–
	Romancheinidae	*Escharella immersa*	–	FJ196144	–	–
	Scrupariidae	*Scruparia chelata*	JN680952	–	–	–
	Triticellidae	*Triticella pedicellata*	–	FJ196150	–	–
Phoronida		*Phoronis ovalis*	GU125758	GU125743	–	–
		*Phoronis vancouverensis*	FJ196118	–	FJ196088	AF342797
		*Phoronopsis harmeri*	AF123308	–	–	–
Brachiopoda		*Argyrotheca cordata*	AF119078	–	–	–
		*Laqueus quadratus*	–	–	AB026505	–
		*Lingula reevii*	AB747096	–	–	–
		*Novocrania anomala*	DQ279934	AY210463	–	–
		*Terebratalia transversa*	–	–	–	AF342802

Sequence data which was generated during this study is indicated with asterisks.

Analyses of genetic distances presented as *P*-values were performed between the freshwater, brackish, and marine bryozoan taxons with members of Brachiopoda and Phoronida as out-groups. Analyses of genetic distances were performed using the Tamura-Nei model with the proposed gamma distribution (TrN + G) in the software package MEGA5 5.2.2.2 (Tamura et al. 2011). Genetic distances were calculated for four sets of nucleotide sequences (18S rRNA: 34 nucleotide sequences with 548 positions; 28S rRNA: 24 nucleotide sequences with 276 positions; COI: 20 nucleotide sequences with 471 positions; and ITS2: 23 nucleotide sequences with 147 positions).

## Results

### Genetic distances

**Figure 2:**
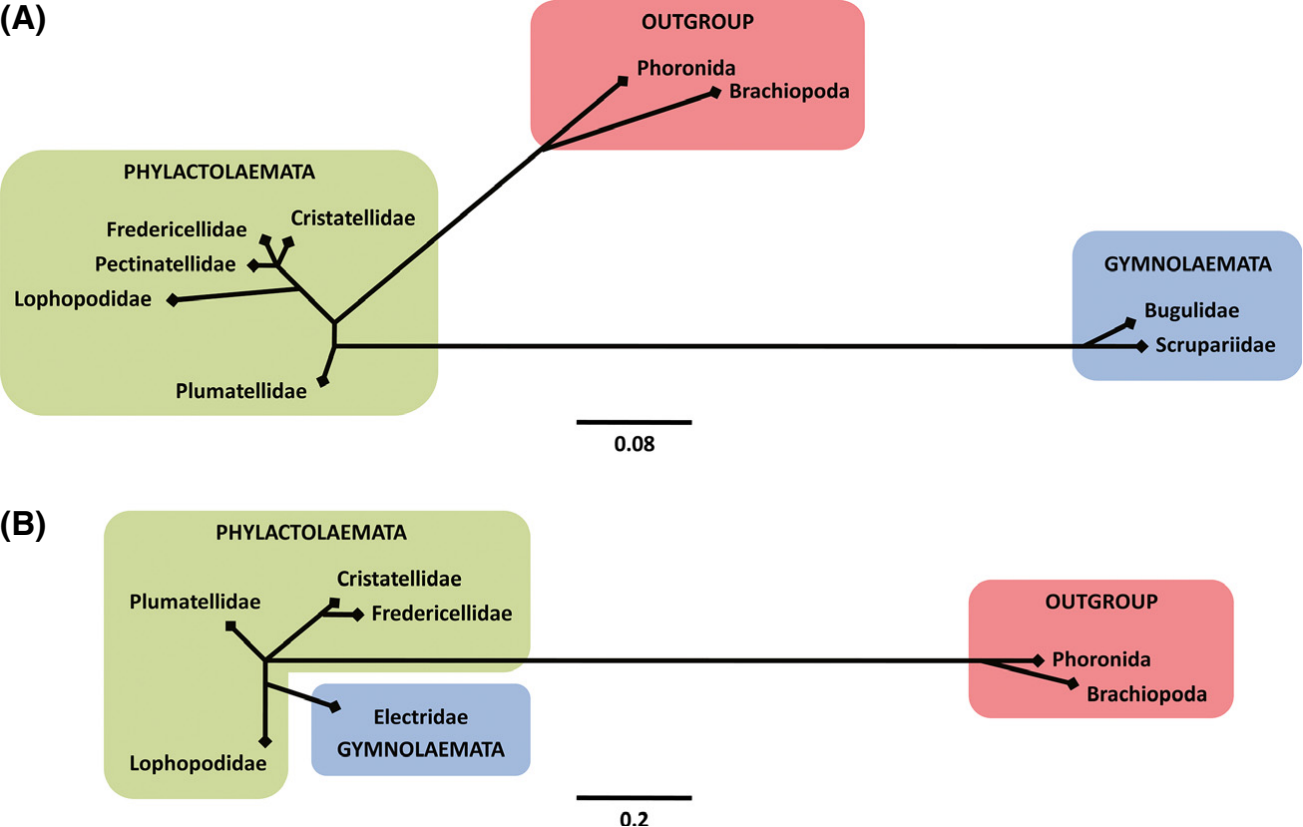
Genetic distances between the Phylactolaemata and Gymnolaemata family groups and out-group members (Phoronida and Brachiopoda) based on 18S (A) and ITS2 sequences (B).

Data on genetic distances show greater similarity of Phylactolaemata freshwater taxons with Brachiopoda and Phoronida out-group taxons than with Gymnolaemata marine taxons (Fig.2A). Considering genetic values and distances between Phylactolaemata freshwater taxons, Gymnolaemata brackish taxons, and out-group taxons, there is a greater genetic similarity between Phylactolaemata freshwater taxons and Gymnolaemata brackish taxons (Fig.2B). Genetic distances between Gymnolaemata and Phylactoalemata taxons show differences regarding the type of the ecosystem which certain Phylactolaemata taxon inhabits. The values are lower if Phylactolaemata taxons inhabit strictly freshwater ecosystem while they are larger if Phylactolaemata taxons inhabit brackish waters.

Distance based on ITS2 sequences between Phylactolaemata freshwater species *Plumatella repens* and Phoronida species *Phoronis vancouverensis* is 1.450, while between *P. repens* and Brachiopoda species *Terebratalia transversa* is 1.637, which indicates a closer genetic relationship between Phylactolaemata and Phoronida phylum. Distance based on 18S sequences between Phoronida species *Phoronopsis harmeri* and Phylactolaemata freshwater species *Hyalinella punctata* is 0.062, while between *P. harmeri* and Gymnolaemata marine species *Bugula plumosa* is 0.193, which indicates a closer genetic relationship of Phylactolaemata with the out-group and the Gymnolamata class as genetically distant. Distance based on 18S sequences between Gymnolaemata marine species *Bugula plumosa* and Phylactolaemata freshwater species *Plumatella repens* is 0.183, while between *B. plumosa* and Phylactolaemata brackish species *Lophopus crystallinus* is 0.192, which indicates a distant genetic relationship of brackish Phylactolaemata species with the Gymnolamata class. Distance based on ITS2 sequences between Gymnolaemata brackish species *Conopeum seurati* and Phylactolaemata freshwater species *Cristatella mucedo* is 0.236 as between *C. seurati* and Phylactolaemata brackish species *Lophopus crystallinus* is 0.089, which indicates a closer genetic relationship of brackish Gymanolaemata species with Phylactolaemata brackish species and distant genetic relationship with Phylactolaemata freshwater species.

### Phylogenetic relationships

**Figure 3:**
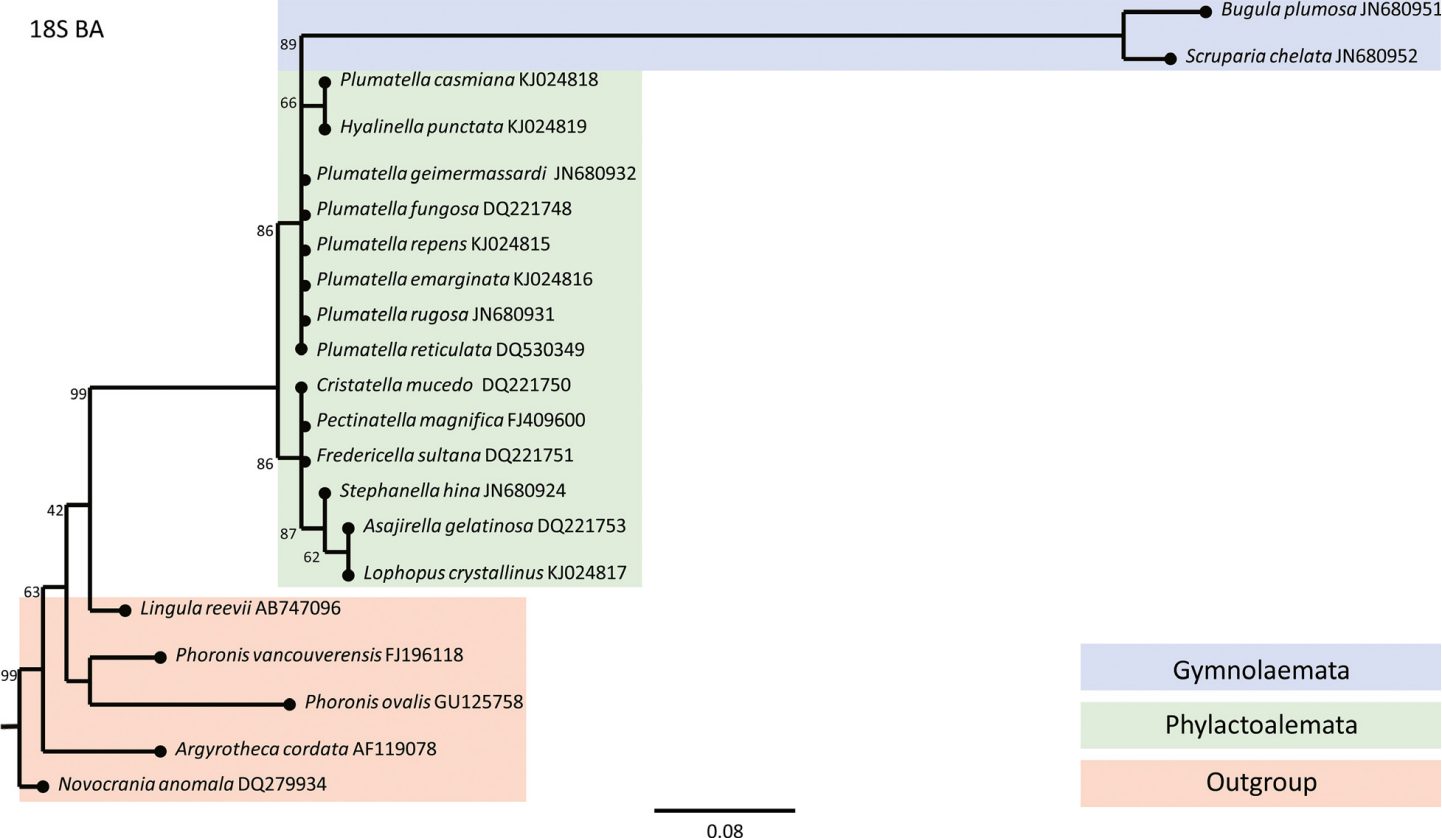
Phylogenetic tree constructed by BA analysis based on 18S nuclear gene that shows the relationships within the Phylactolaemata class and relationship toward the Gymnolaemata class and out-group members. End of the each branch indicates the habitat where the species occurs.

**Figure 4:**
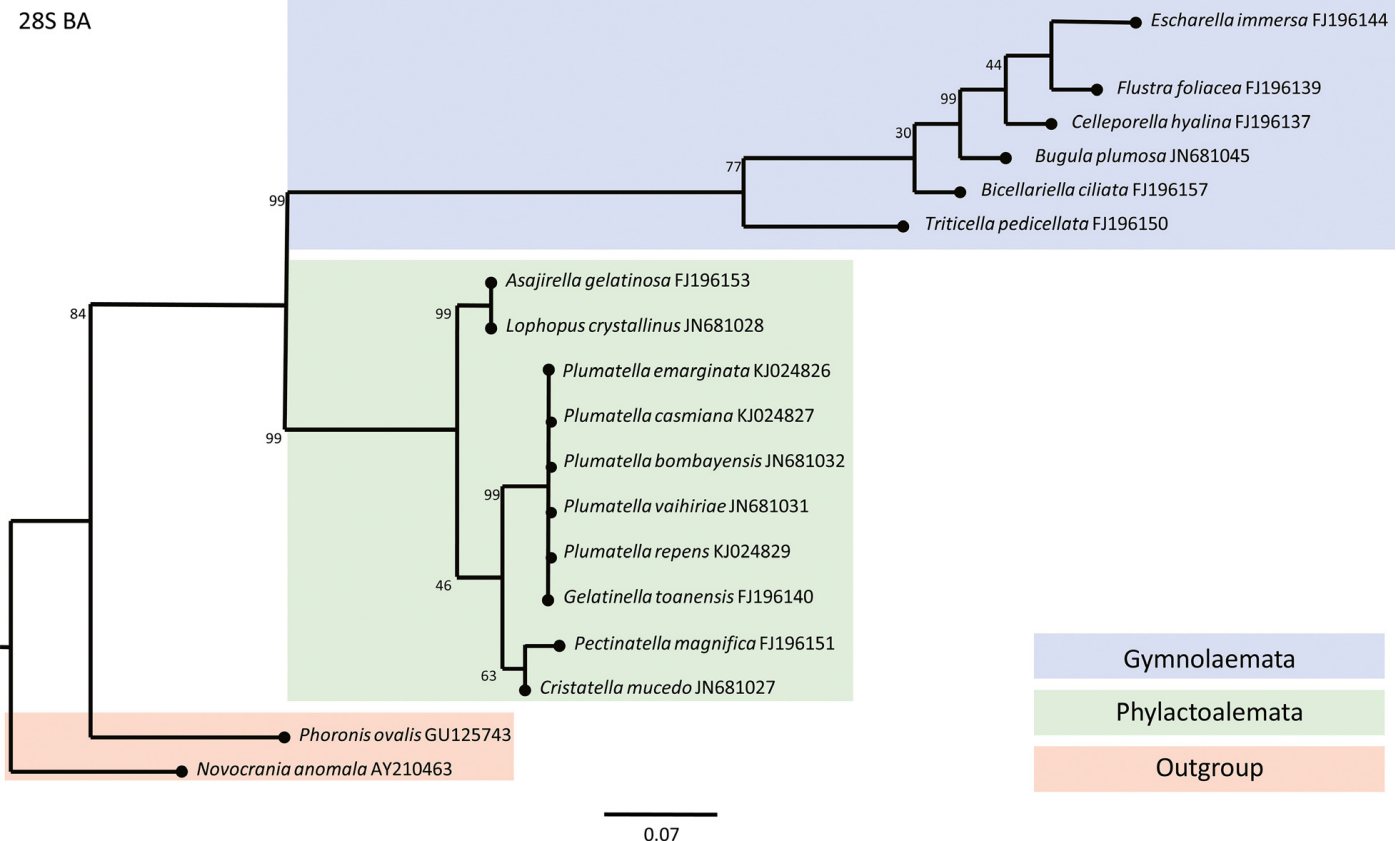
Phylogenetic tree constructed by BA analysis based on 28S nuclear gene that shows the relationships within the Phylactolaemata class and relationship toward the Gymnolaemata class and out-group members. End of the each branch indicates the habitat where the species occurs.

**Figure 5:**
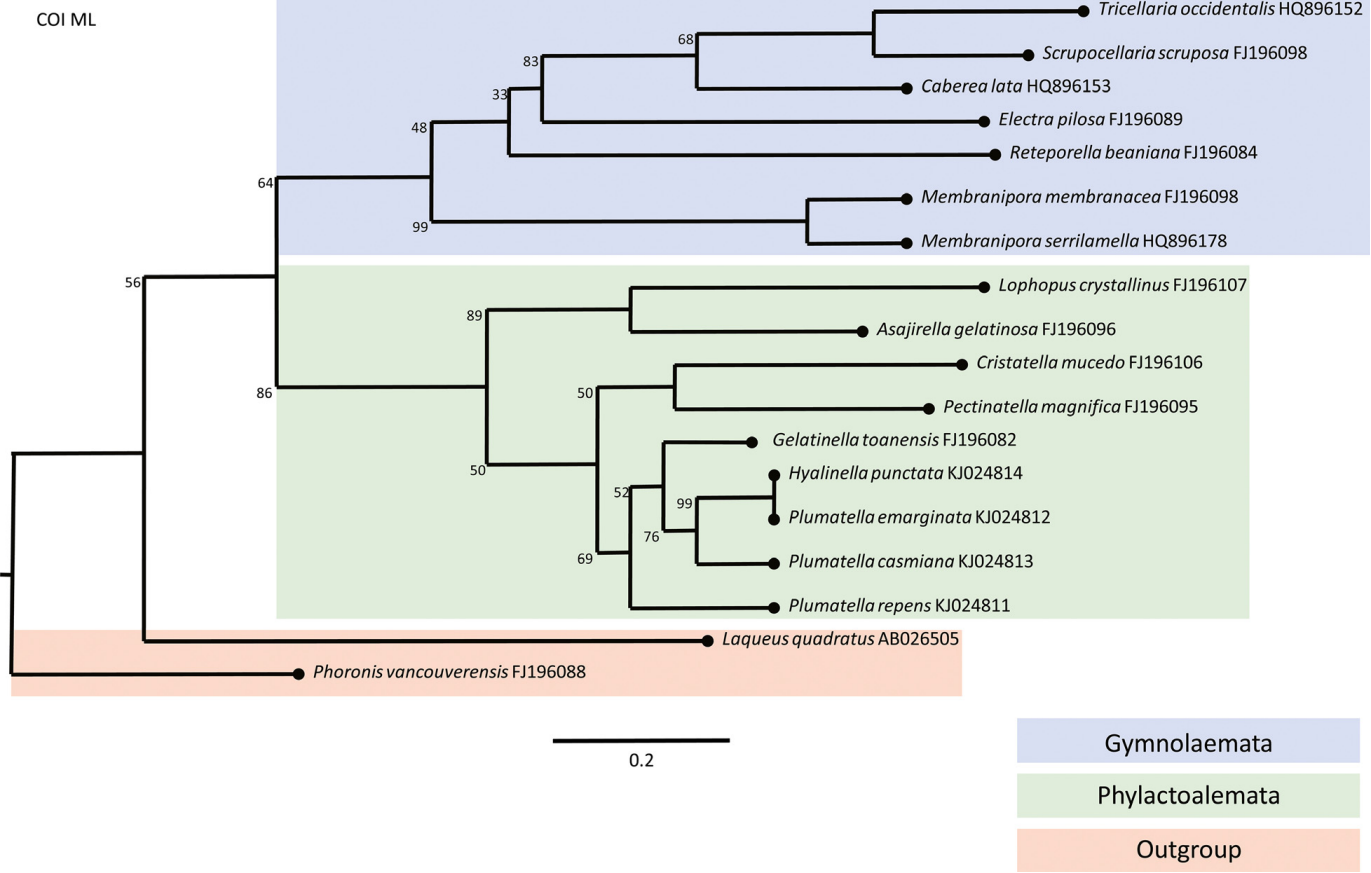
Phylogenetic tree constructed by ML analysis based on COI mitochondrial gene that shows the relationships within the Phylactolaemata class and relationship toward the Gymnolaemata class and out-group members. End of the each branch indicates the habitat where the species occurs.

**Figure 6:**
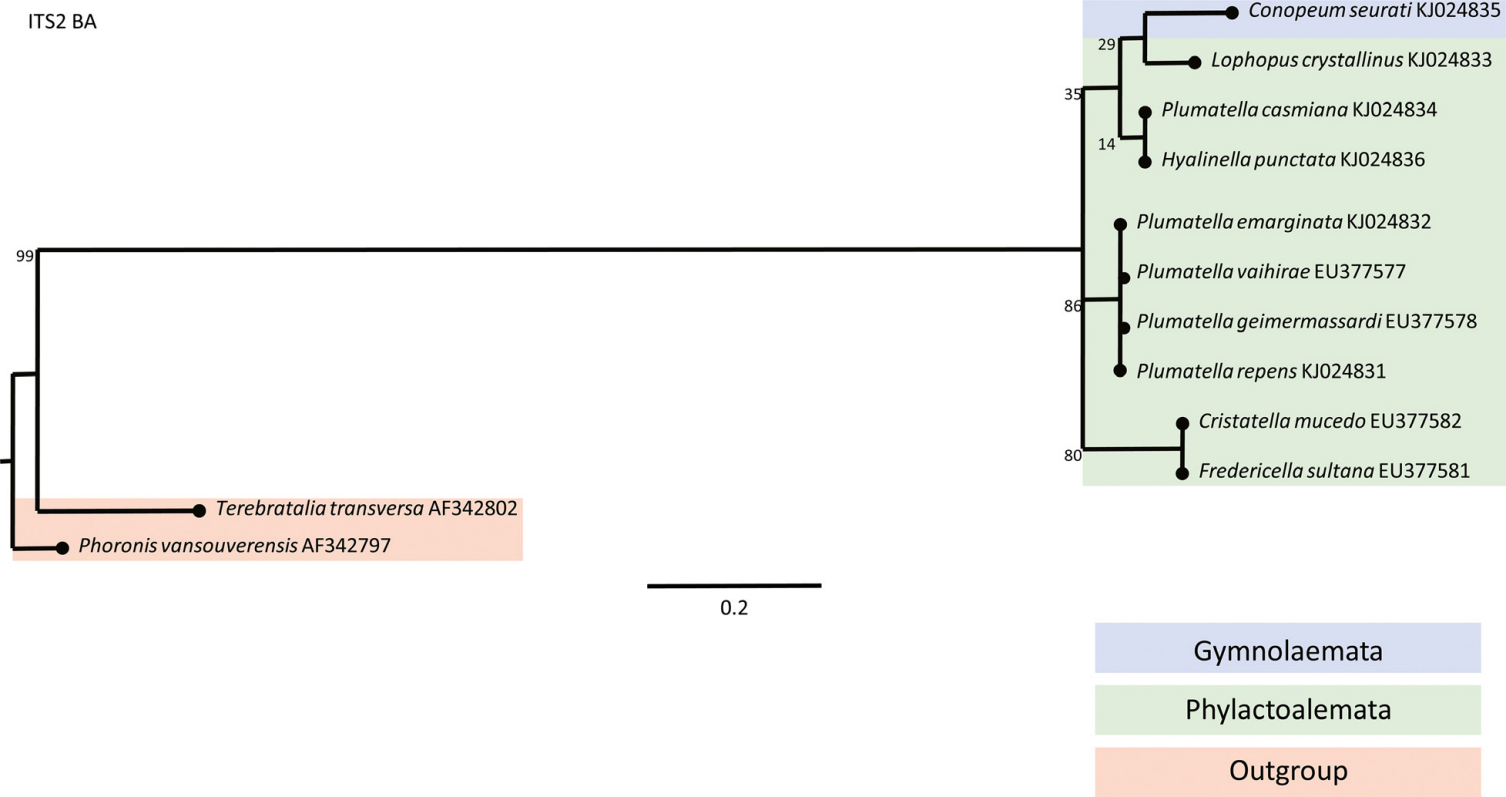
Phylogenetic tree constructed by BA analysis based on ITS2 nuclear gene that shows the relationships within the Phylactolaemata class and relationship toward the Gymnolaemata class and out-group members. End of the each branch indicates the habitat where the species occurs.

Taxons of Bryozoa which inhabit brackish waters are situated on the phylogenetic tree in two genetically distant classes of bryozoans: Phylactolaemata (*Lophopus crystallinus*) and Gymnolaemata (*Conopeum seurati*). Due to previous research, monophyletic bryozoan origin is proven by assembling phylogenetic trees with members of two classes of bryozoans (Phylactolaemata and Gymnolaemata) with members of Phoronida and Brachiopoda as out-groups. Phylogenetic studies have shown a closer relationship of Phylactolaemata taxons to Phoronida group than of Phylactolaemata taxons to the Gymnolaemata group. Constructed phylogenetic trees based on the sequences of 18S (Fig.3), 28S (Fig.4), and COI (Fig.5) gene as well as ITS2 region (Fig.6) confirmed that the Lophopodidae family is a sister group to Cristatellidae, Pectinatellidae, Fredericellidae and Plumatellidae family group. The Cristatellidae and Pectinatellidae families show closer relatedness and form a sister group to the Plumatellidae family. Phylogenetic trees show minor differences in morphology because the trees were not always constructed with the identical taxons regarding the four used phylogenetic markers. The divergence of branches and nodes on the trees varies with four displayed trees because the markers for gene COI and ITS2 region are much more specific in constructing phylogenetic trees and are used in projecting the position of species in systematic. Markers for 18S and 28S genes are suitable for constructing phylogenetic trees on higher systematic levels.

Regardless of the lack of fossil evidences of freshwater and brackish taxons to support this model, we propose the theory which can be significant, taking into account the fact that the evolutionary age is confirmed by the molecular phylogenetic analysis and shown in the phylogenetic trees. The model is based on the fact that the last common ancestor of today's Phylactolaemata bryozoans first evolved in marine environment and afterward, during its evolution, occupied freshwaters as a new habitat.

The position of Phylactolaemata taxons on the phylogentic tree with other members of recent taxons of bryozoans and out-groups represents the Phylactolaemata group as the base, while the Gymnolaemata group separated independently during their collective evolutionary history. Taking this fact into consideration, today we speak of Phylactolaemata as freshwater and brackish bryozoans. Later in evolutionary time, they were separated from classes Gymnolaemata and Stenolaemata whose representatives are present predominantly as marine species and whose fossils we can discover because of the mineral composition of their colonies. Stenolaemata are bryozoans with exclusively marine taxons, and they represent evolutionarily the youngest group within the bryozoans. Gymnolaemata are bryozoans with marine and brackish taxons, and they are evolutionarily younger than Phylactolaemata. The obtained model of bryozoan evolutionary development is comparable with theories about the monophyletic origin of bryozoans from previous authors.

## Discussion

The calculated genetic distances as well as the positions on the phylogenetic trees reconstructed on the basis of the two aforementioned analyses and the analyzed four phylogenetic markers suggest that the Lophopodidae family is evolutionarily the oldest family in the Phylactolaemata class. From the phylogenetic view, it is clear that the Lophopodidae family separated independently in the evolutionary history of freshwater taxons from all other members of Plumatellida genus of Phylactolaemata class. This fact supports the theory that the brackish species from Phylactolaemata class separated earlier in the evolutionary history and independently conquered brackish waters. An example of that event is brackish Phylactolaemata species *Lophopus crystallinus* from the Lophopodidae family.

**Figure 7:**
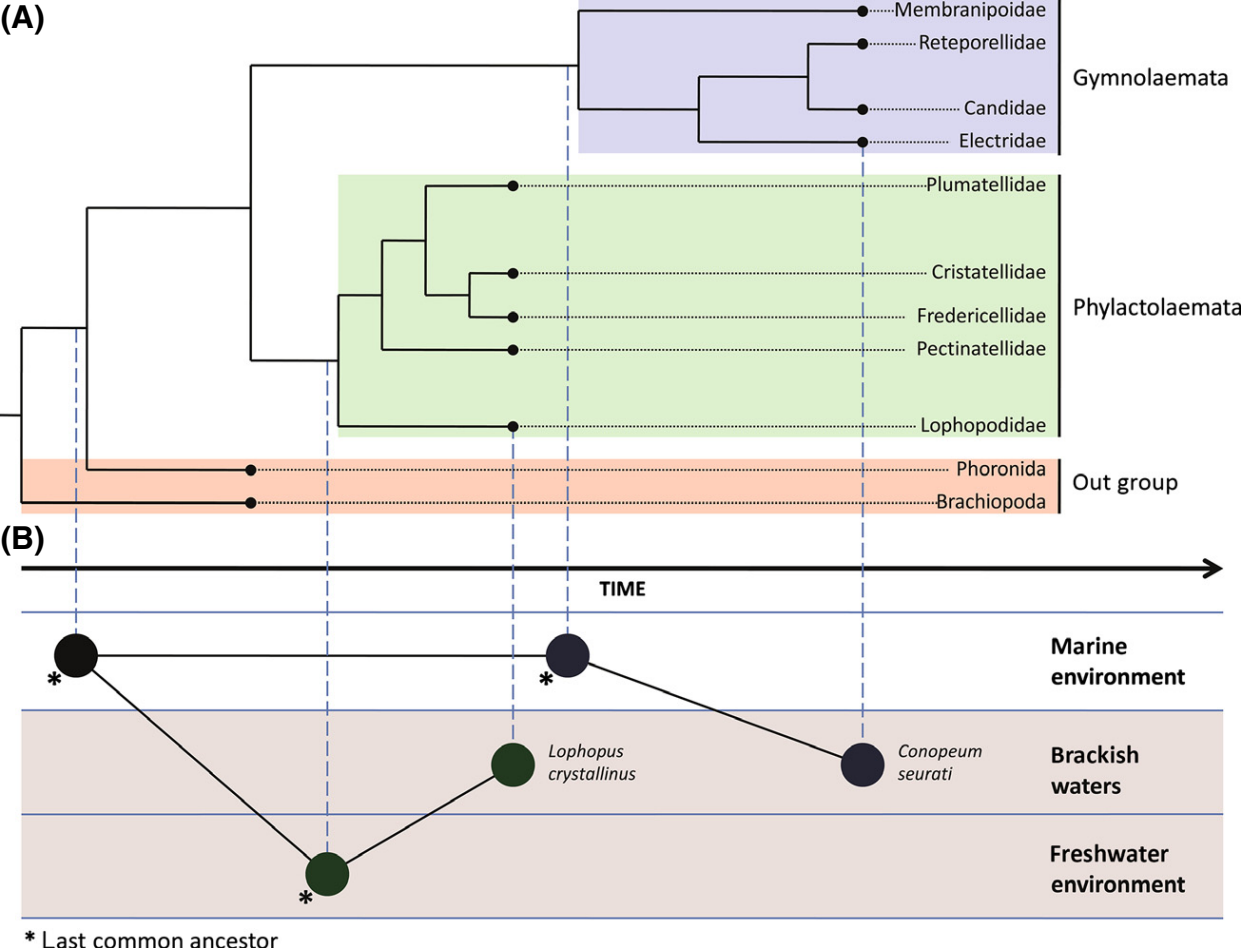
Graphics of general genetic relations of Phylactolaemata and Gymnolaemata taxons with out-group members (A). Position of a certain taxon from the tree is associated with the proposed model of double colonization of freshwater ecosystems by the bryozoan group during their evolutionary time (B).

The proposed phylogeny of Bryozoa categorizes freshwater and brackish bryozoans from the Phylactolaemata class as the radix group and evolutionarily the oldest while taxons from the Gymnolaemata class as evolutionarily younger (Jebram 1973; Boardman et al. 1983; Ax 2001). Today's understanding of Phylactolaemata bryozoans as the radix group of all bryozoans must be interpreted together with the fact that the common ancestors of all bryozoans were a marine organism at the time of separation from the closest related group (Phoronida and Brachiopoda) when it colonized freshwater ecosystems. Therefore, we propose a theory of colonization of freshwater ecosystems on two occasions by the same group of animals during their evolutionary history (Fig.7). The ancestor of all bryozoans that lived in the marine environment colonized the freshwater ecosystems where the group now known as freshwater bryozoan taxons from the Phylactolaemata class was formed. Over the evolutionary history, there was a development of certain groups of bryozoan taxons that colonized the marine habitats and formed the Gymnolaemata and Stenolaemata classes. Independently, segregation of brackish taxons from the Phylactolaemata class occurred. The second round of colonization of freshwater environment was conducted by Gymnolaemata taxons whose representatives are present species which inhabit brackish water. An example for that event is the brackish Gymnolaemata species *Conopeum seurati* from the Electridae family.

The model is proposed together with phylogenetic facts about the development of groups within Bryozoa phylum with the focus of phylogenetic relationship of Phylactolaemata and Gymnolaemata taxons and their ecological status. As evolutionarily the oldest, the Phylactolaemata bryozoans with present dominant freshwater and brackish taxons were the first of bryozoans who lived in freshwater environment. As evolutionarily younger, the Gymnolaemata bryozoans with present dominant marine taxons adapted to freshwater environment for the second time in their collective evolution, and the proof of that event is the brackish taxon from this group.

Research shows genetic links between brackish and marine bryozoan species and analyzed evolutionary models show that the development of marine groups occurred parallel with colonization of freshwater ecosystems. Great genetic similarity between the Lophopodidae family members and marine bryozoans taxons projected through small genetic distance is associated with the environmental factor and characterizing some members of the Lophopodidae family as brackish taxons. As evolutionarily oldest group of freshwater bryozoans, members of the Lophopodidae family which inhabit brackish and freshwater environments could be a new transitional form, this time from freshwater to marine ecosystems.

## Data Archiving Statement

Data for this study are available at the Genbank link: http://www.ncbi.nlm.nih.gov/genbank/
